# Trends in dispensing and utilisation of sustained-release tapentadol in Australia.

**DOI:** 10.23889/ijpds.v7i3.1978

**Published:** 2022-08-25

**Authors:** Ximena Camacho, Andrea Schaffer, Nicholas Buckley, Nicole Pratt, Jonathan Brett, David Henry, Sallie-Anne Pearson

**Affiliations:** 1 Centre for Big Data Research in Health, Faculty of Medicine, UNSW Sydney; 2 Biomedical Informatics and Digital Health, The University of Sydney; 3 University of South Australia; 4 Bond University

## Objectives

Tapentadol is a µ-opioid agonist, moderate noradrenaline reuptake inhibitor (NRI) and very weak serotonin reuptake inhibitor. The sustained release (SR) formulation is indicated for relief of chronic moderate to severe pain. We examined utilisation trends and concordance with prescribing guidelines of prescription SR tapentadol in Australia between 2014-2021.

## Approach

We used a 10% sample of Australian Pharmaceutical Benefits Scheme dispensing data linked to death records. We assessed incidence, prevalence, and trends in monthly dispensed prescriptions between June 2014 (date of public subsidy) and September 2021. We defined incident users in adults aged ≥18 years as no tapentadol dispensings in the previous year. We assessed characteristics of initiators and patterns of utilization in the year following initiation, including prescription strengths, concurrent dispensing of tapentadol and medicines with known interactions or contraindications, and switching to other opioids.

## Results

4,883,840 prescriptions of tapentadol were dispensed during the study period. Total monthly dispensed prescriptions increased from 2,040 (June 2014) to 91,300 (September 2021). The lowest strength formulations (50mg, 100mg) comprised nearly three quarters of all dispensed prescriptions (3,628,400; 74.3%). We identified 66,334 new episodes of tapentadol use. Incidence rose from 0.8/1000 population in 2014 to a high of 8.4/1000 in 2019, dropping to 5.5/1000 in 2020. Most people initiating tapentadol were aged 45-84 years (48,464; 73.1%) and female (37,709; 56.9%). More than half (38,157; 57.5%) did not receive a second tapentadol dispensing within 90 days of initiation. 10,868 (16.4%) were dispensed a non-opioid medicine with known interaction on the same day as initiation, and 23,205 (35.0%) were concurrently dispensed tapentadol and other opioids.

## Conclusion

The only approved subsidized indication is tapentadol SR for chronic pain. However, the very high prevalence of single dispensings of tapentadol SR suggests it may be being used off-label for acute pain instead of tapentadol immediate-release; this would be less safe, less effective and also against Australian therapeutic guidelines.

